# The Handbook of Practical Receipts for Every Day Use

**Published:** 1857-12

**Authors:** 


THE HANDBOOK OF PRACTICAL RECEIPTS FOR EVERYDAY USE: a Manual for the Chemist, Druggist, Medical Practitioner, Manufacturer, and Heads of Families; comprising the Officinal Medicines, their uses and modes of preparation, and Formulæ for Trade Preparations, Mineral Waters, Powders, Beverages, Dietetic Articles, Perfumery, Cosmetics, etc.; a Glossary of Terms used in Chemistry and Medicine, including old names, contractions, vulgar and scientific denominations, with a copious index to all the preparations. By THOMAS F. BRANSTON. First American, from the second London edition. Philadelphia: Lindsay & Blackiston. 1857. Pages 307.

"This manual is offered in the hope that it will prove a useful companion to those for whom it is intended." (Preface.)

If it is useful to those for whom it is intended, it will be useful to everybody, for nearly everybody is included in the title. This is the kind of book to sell on a large scale. We would refer all who desire to purchase one of them to Messrs. Keen and Lee, Lake street, Chicago, and assure the people that if they will purchase one, they will not need any more doctors to cure them, preachers to administer the last sacrament to them, nor lawyers to settle up their estate.

